# Supplementary material for: Phylogenetic Analyses of Sites in Different Protein Structural Environments Result in Distinct Placements of the Metazoan Root
Source: Biology (Basel). 2020 Mar 28;9(4):64. doi: 10.3390/biology9040064 (PMC7235752; doi:10.3390/biology9040064)
Supplement: Supplementary file 1 [file biology-09-00064-s001.zip › Pandey_Braun_2020_supplementary_files/PB20_Supplementary_Figures.pdf]

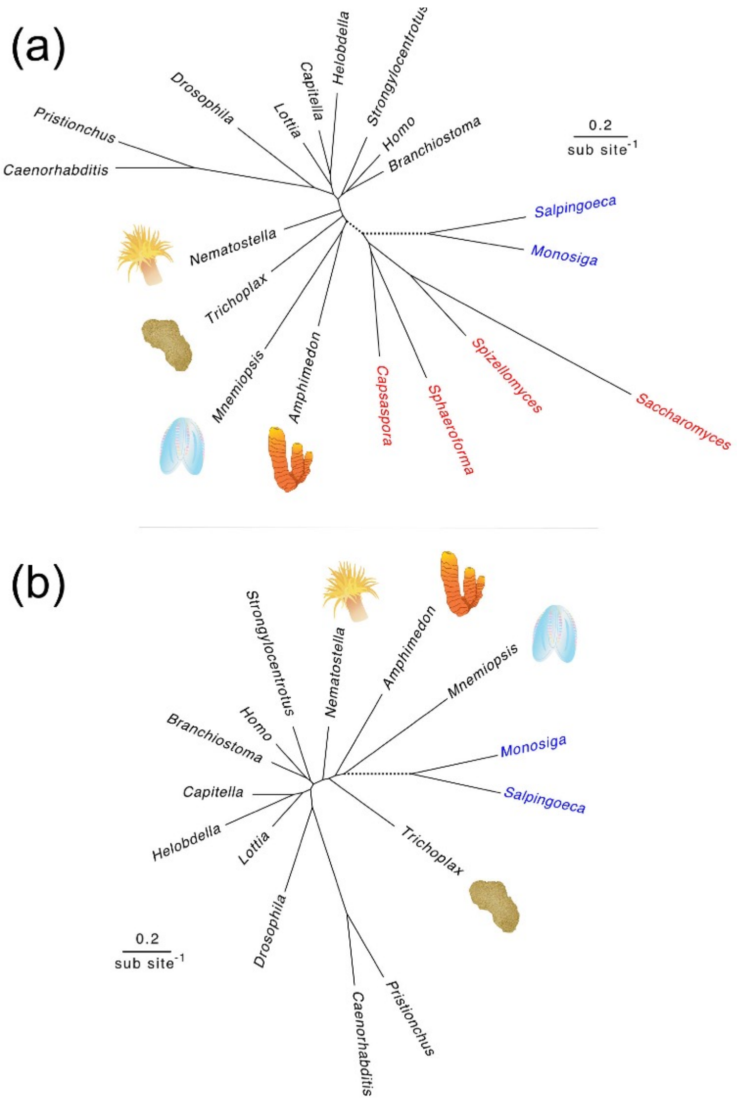

Supplementary Figure S1. Estimates of metazoan phylogeny based on buried residues presented as unrooted phylograms. Outgroup taxa are emphasized using blue (Choanozoa) and red (other outgroups). (**a**) Complete taxon set. (**b**) Reduced (Apoikozoa only) taxon set. We have indicated the branch separating the choanozoan outgroups from the ingroup using a dashed line. The tree for the complete taxon set shows that the other outgroup taxa almost bisects the branch to Choanozoa. The drawings used to illustrate taxa are identical to those used to illustrate the major clades in Figure 1. We have limited these figures to the trees based on buried sites; nexus and newick format treefiles for all subsets of the data are available in other supplementary files.

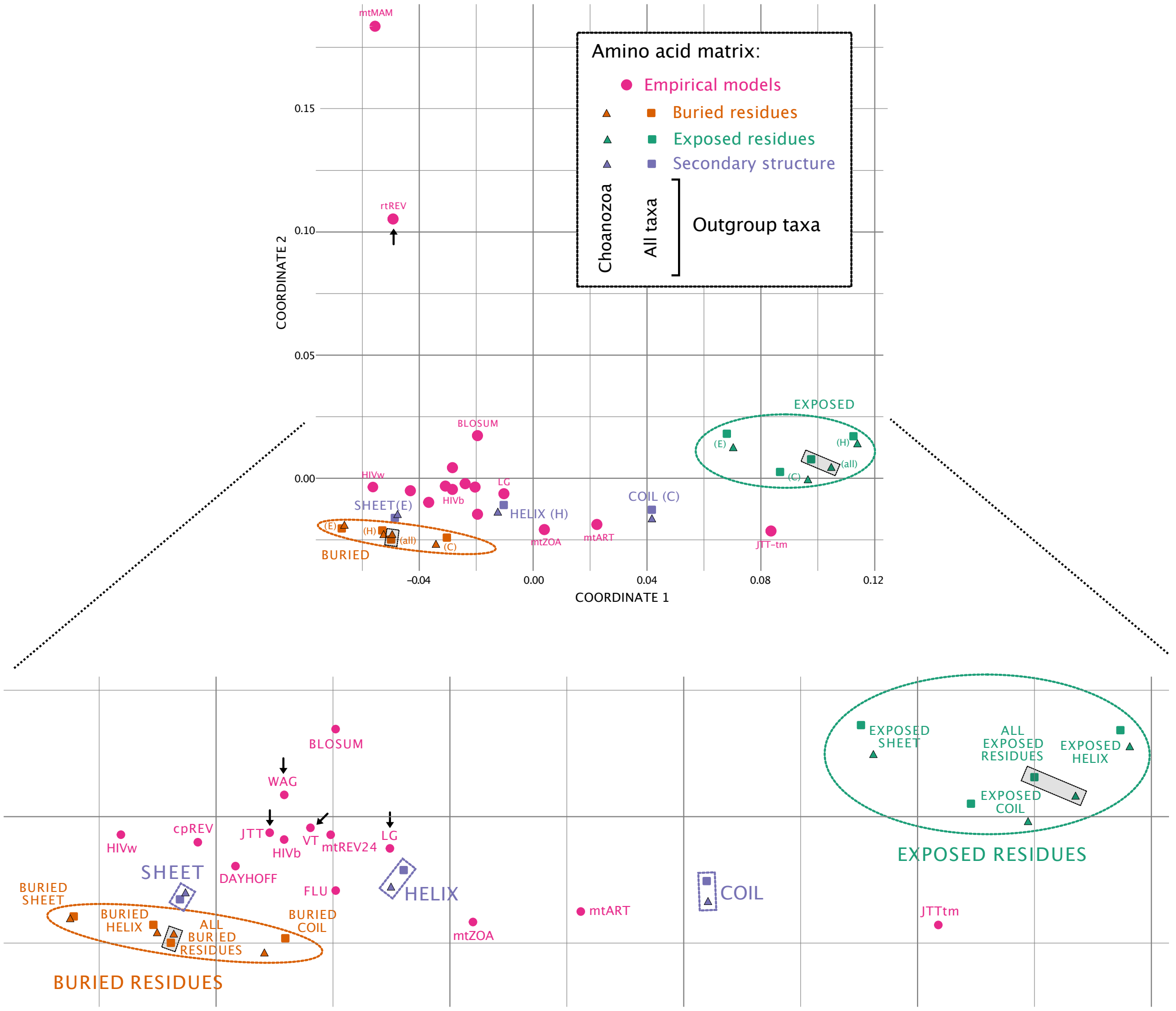

Supplementary Figure S2. Multidimensional scaling plot based on Euclidean distances among amino acids exchange rate matrices. This plot expands fig. 4 to allow us to label all standard empirical models (pink circles); small arrows indicate the empirical models used in this study. Squares and triangles indicate rate matrices estimated using either all taxa (squares) or the reduced taxon set limited to choanozan outgroups (the Apoikozoa taxon set). Green and orange dotted ovals enclose clusters of rate matrices for exposed and buried residues, respectively; rate matrices based on all exposed or buried sites are emphasized by shaded boxes within those ovals. Rate matrices based on exposed or buried sites separated into subsets based on secondary structure are paired and labeled. Purple rectangular boxes indicate the three groups of rate matrices based on secondary structure (helix, sheet, and coil) without separating those residues into exposed and buried subsets.

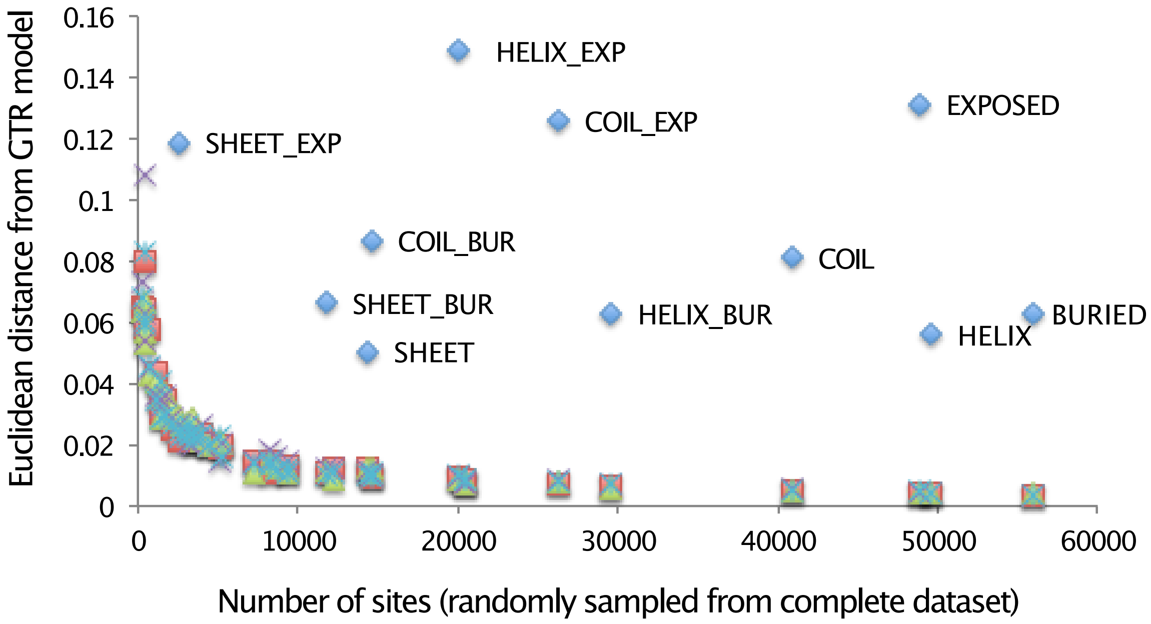

Supplementary Figure S3. Euclidean distance between "grand" GTR rate matrix parameters (i.e., the rate matrix parameters estimated using the GTR model with the complete FRG dataset) and GTR rate matrix parameters estimated using subsets of the data. Rate matrix parameters optimized on sites defined using protein structure are presented as blue diamonds. The other points represent distances between the grand GTR model and rate matrix parameter estimates optimized using random samples (ranging in size from 500-55,000 aligned amino acid sites) that were drawn from the concatenated FRG dataset. For each data subset size, we generated 10 random samples; the distance between the rate matrix for structurally defined sites and the grand GTR model always exceeds the distance for random samples of sites of comparable size.
