# Supplementary material for: Phylogenetic Analyses of Sites in Different Protein Structural Environments Result in Distinct Placements of the Metazoan Root
Source: Biology (Basel). 2020 Mar 28;9(4):64. doi: 10.3390/biology9040064 (PMC7235752; doi:10.3390/biology9040064)
Supplement: Supplementary file 1 [file biology-09-00064-s001.zip › Pandey_Braun_2020_supplementary_files/PB20_Supplementary_File_S4.zip!/PB20_Supplementary_File_S4/CAT_model_profile_examples.docx]

Examples CAT model profiles

Profile 21 for the C60 model of Le et al. (2008) has the narrowest profile. It appears to capture conserved tryptophan residues; only three other amino acids have equilibrium frequencies > 0.01. It has an effective alphabet size of 1.929 (calculated using equation 5 from Pollock et al. 2012).

The amino acids are sorted by equilibrium frequency and the cumulative frequency is presented to show that the equilibrium frequencies of the three aromatic amino acids sum to a value > 0.95.

| **Amino Acid** | **Equilibrium Frequency (π)** | **Cumulative Frequency** |
| --- | --- | --- |
| W | 0.86404369 | 0.86404369 |
| F | 0.0513377 | 0.9153814 |
| Y | 0.03909126 | 0.95447265 |
| L | 0.01274208 | 0.96721474 |
| R | 0.00606743 | 0.97328217 |
| G | 0.00355796 | 0.97684013 |
| V | 0.00331373 | 0.98015386 |
| C | 0.00290594 | 0.9830598 |
| H | 0.00276305 | 0.98582285 |
| M | 0.00256714 | 0.98838998 |
| A | 0.00170034 | 0.99009032 |
| Q | 0.00164242 | 0.99173274 |
| T | 0.00140331 | 0.99313605 |
| P | 0.00135367 | 0.99448972 |
| I | 0.00122912 | 0.99571884 |
| S | 0.00118717 | 0.99690601 |
| E | 0.00117317 | 0.99807918 |
| D | 0.00107115 | 0.99915033 |
| K | 0.00042738 | 0.99957771 |
| N | 0.00042229 | 1 |

An example of a wider profile, with an effective alphabet size similar to the median for the C60 model, is provided on the next page.

References

Le, S.Q.; Lartillot, N.; Gascuel, O. Phylogenetic mixture models for proteins. *Philos. Trans. Royal Soc. B*, **2008**, *363*, 3965-3976.

Pollock, D.D.; Thiltgen, G.; Goldstein, R.A. Amino acid coevolution induces an evolutionary Stokes shift. *Proc. Natl. Acad. Sci. U. S. A.*, **2012**, *109*, E1352-E1359.

Profile 31 for the C60 model from Le et al. (2008) is a more typical profile, with an effective alphabet size (8.729) near the median for the C60 model. It appears to capture sites dominated by moderately sized polar residues, like serine, asparagine, and threonine.

| **Amino Acid** | **Equilibrium Frequency (π)** | **Cumulative Frequency** |
| --- | --- | --- |
| S | 0.28298026 | 0.28298026 |
| N | 0.27835228 | 0.56133253 |
| T | 0.08603171 | 0.64736424 |
| A | 0.06016412 | 0.70752836 |
| G | 0.05430807 | 0.76183643 |
| H | 0.05315235 | 0.81498878 |
| D | 0.03371888 | 0.84870766 |
| C | 0.0315067 | 0.88021436 |
| Q | 0.0210646 | 0.90127896 |
| R | 0.01619952 | 0.91747848 |
| K | 0.01395984 | 0.93143832 |
| Y | 0.01348495 | 0.94492326 |
| V | 0.01009536 | 0.95501862 |
| M | 0.00882987 | 0.96384848 |
| P | 0.00758343 | 0.97143192 |
| L | 0.00706981 | 0.97850173 |
| I | 0.00706508 | 0.98556681 |
| F | 0.00695259 | 0.9925194 |
| E | 0.00598395 | 0.99850335 |
| W | 0.00149666 | 1 |
